# Synthesis of [^18^F]FMISO, a hypoxia-specific imaging probe for PET, an overview from a radiochemist’s perspective

**DOI:** 10.1186/s41181-023-00190-7

**Published:** 2023-03-10

**Authors:** Torsten Kniess, Jörg Zessin, Peter Mäding, Manuela Kuchar, Oliver Kiss, Klaus Kopka

**Affiliations:** 1 Institute of Radiopharmaceutical Cancer Research, Helmholtz-Zentrum Dresden-Rossendorf, Bautzner Landstraße 400, 01328 Dresden, Germany; 2 Faculty of Chemistry and Food Chemistry, School of Science, Technische Universität Dresden, Mommsenstraße 4, 01069 Dresden, Germany; 3 National Center for Tumor Diseases (NCT) Dresden, University Hospital Carl Gustav Carus, Fetscherstraße 74, 01307 Dresden, Germany; 4 German Cancer Consortium (DKTK), Partner Site Dresden, Fetscherstraße 74, 01307 Dresden, Germany

**Keywords:** [^18^F]FMISO, [^18^F]fluoromisonidazole, Automated radiosynthesis, FASTlab

## Abstract

**Background:**

[^18^F]fluoromisonidazole ([^18^F]FMISO, 1*H*-1-(3-[^18^F]fluoro-2-hydroxypropyl)-2-nitroimidazole) is a commonly used radiotracer for imaging hypoxic conditions in cells. Since hypoxia is prevalent in solid tumors, [^18^F]FMISO is in clinical application for decades to explore oxygen demand in cancer cells and the resulting impact on radiotherapy and chemotherapy.

**Results:**

Since the introduction of [^18^F]FMISO as positron emission tomography imaging agent in 1986, a variety of radiosynthesis procedures for the production of this hypoxia tracer has been developed. This paper gives a brief overview on [^18^F]FMISO radiosyntheses published so far from its introduction until now. From a radiopharmaceutical chemist’s perspective, different precursors, radiolabeling approaches and purification methods are discussed as well as used automated radiosynthesizers, including cassette-based and microfluidic systems.

**Conclusion:**

In a GMP compliant radiosynthesis using original cassettes for FASTlab we produced [^18^F]FMISO in 49% radiochemical yield within 48 min with radiochemical purities > 99% and molar activities > 500 GBq/µmol. In addition, we report an easy and efficient radiosynthesis of [^18^F]FMISO, based on in-house prepared FASTlab cassettes, providing the radiotracer for research and preclinical purposes in good radiochemical yields (39%), high radiochemical purities (> 99%) and high molar activity (> 500 GBq/µmol) in a well-priced option.

## Background

The molecular imaging technology positron emission tomography (PET), nowadays implemented in hybrid PET/computed tomography (PET/CT) and PET/magnetic resonance imaging (PET/MRI) systems, is a modern, more and more evolving approach for non-invasive imaging of hypoxia. Among the radiotracers that are selectively targeting hypoxic conditions in cells, [^18^F]fluoromisonidazole ([^18^F]FMISO, 1*H*-1-(3-[^18^F]fluoro-2-hydroxypropyl)-2-nitroimidazole), an ^18^F-labeled nitroimidazole, is one of the most established ones (Xu et al. 2017). Since hypoxia is prevalent in a manifold of solid tumors such as head-and-neck cancers, glioblastoma, gastrointestinal tumours, lung and breast cancer, [^18^F]FMISO is in clinical application since 1992 (Koh et al. 1992), and therefore for more than 30 years. Hypoxic tumors in general are associated by an unfavorable prognosis, not only due to a higher radioresistance, but also by showing more recurrences after surgery or chemotherapy, according to a more aggressive tumor biology and clinical phenotype (Vaupel et al. 2001). Hypoxia is therefore considered to be a useful parameter for pre-therapeutic or intra-therapeutic stratification in radiochemotherapy trials, identifying patients with a high risk of recurrence (Thorwarth et al. 2019; Bandurska-Luque et al. 2019; Lee et al. 2015; Zips et al. 2012).

Since the clinical application of [^18^F]FMISO, more than 600 scientific related papers—as indexed by PubMed® in December 2022—underline the impact of this nitroimidazole based imaging probe. A review on selected ^18^F- and the ^123^I-radiolabeled nitroaromatics, the counterparts for single photon emission computed tomography (SPECT), that directly exploit the bioreductive environment in hypoxic cells was published in 2015 (Kumar et al. 2015). The present mini﻿-review focuses on the manufacturing and purification strategies of [^18^F]FMISO from a radiopharmaceutical chemist’s view including manual and automated as well microfluidic and cassette-based synthesis approaches. Finally, we describe herein a low-cost radiosynthesis of [^18^F]FMISO based on in-house prepared cassettes.

From a radiopharmaceutical chemist’s perspective starting in 1986 with laborious radiolabelling an purification steps with low radiopharmaceutical yields it took 20 years, to develop fully automated radiolabeling procedures, which provide [^18^F]FMISO in reproducible high yields as well as in good radiopharmaceutical quality according to Ph. Eur. Monograph 2459.

**Scheme 1.**
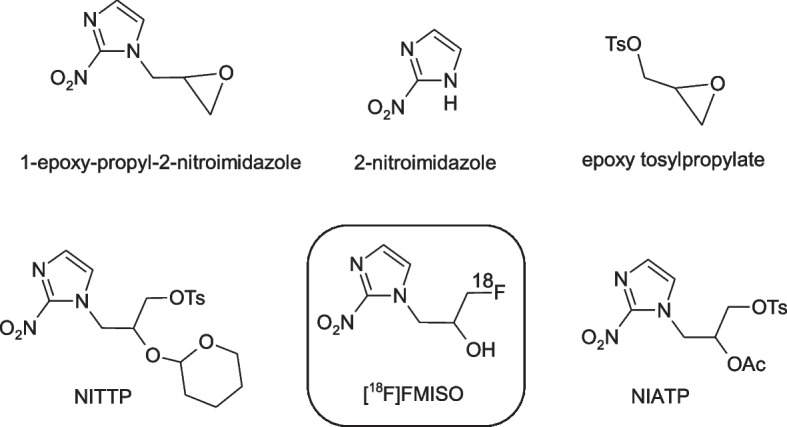
[^18^F]FMISO and radiolabeling precursors

An overview on [^18^F]FMISO manufacturing methods is given in Table 1. The chemical structures of the different radiolabeling precursors that can be used are shown in Scheme 1. The first ever reported radiosynthesis of [^18^F]FMISO was published by Jerabek and Welch (Jerabek et al. 1986). They introduced a number of nitroimidazoles as potential “biomarkers” for hypoxic cells, among others [^18^F]FMISO. The radiosynthesis of [^18^F]FMISO was based on the ring opening reaction of 1-epoxypropyl-2-nitroimidazole (Scheme 1) with tetrabutylammonium [^18^F]fluoride (TBA[^18^F]F) and yielded the desired radiotracer after a lengthy procedure in 1% radiochemical yield only. However, the obtained activity amount was sufficient for performing first biodistribution studies with encouraging results. In 1989 the same group presented an improved radiosynthesis, starting from the epoxypropyl tosylate precursor (Scheme 1), that was radiolabeled with potassium [^18^F]fluoride in acetonitrile to form epi[^18^F]fluorohydrin. This intermediate was coupled with 2-nitroimidazole to obtain [^18^F]FMISO in 12% yield after HPLC purification and formulation (Hwang et al. 1989). The same radiolabeling strategy was applied by Grierson et al., resulting in 40% radiochemical yield [^18^F]FMISO, but with an overall longer radiosynthesis time (140 min vs. 90 min) (Grierson et al. 1989). In the meantime the epi[^18^F]fluorohydrin based radiosynthesis was further improved by using microwave based heating and remote control. Thereby the radiosynthesis time was reduced to 70 min yielding 40% of purified [^18^F]FMISO (Mccarthy et al. 1993). However, the epi[^18^F]fluorohydrin method was still a two-step radiolabeling process which is not optimal.

**Table 1:** Summary of [^18^F]FMISO manufacturing methods,

Precursor	Radio synthesis method	Purify-cation	Radio synthesis time [min]	Yield [#]	Radio-chemical purity	Molar activity [GBq/µmol]	Reference
1-Epoxy-propyl-2-nitroimidazole	Manual	HPLC	n.d	1% RCY	n.d	n.d	Jerabek t al. 1986
Epoxypropyl tosylate, 2-nitro-imidazole	Manual	HPLC	90	7–12% AY	n.d	14,8	Hwang et al. 1989
Epoxypropyl tosylate, 2-nitro-imidazole	Manual	HPLC	140	40% RCY	n.d	24	Grierson et al. 1989
Epoxypropyl tosylate, 2-nitro-imidazole	Semi-automated	HPLC	70	40% AY	> 98%	n.d	Mccarthy et al. 1993
NITTP	Manual	SPE	45–50	55–80% RCY	n.d	22	Lim et al. 1993
NIATP	Manual	SPE	60–70	12–18% AY	> 99%	74	Cherif et al. 1994
NITTP	Manual	HPLC	5–80	60–86% AY	n.d	n.d	Patt et al. 1999
Epoxypropyl tosylate, 2-nitro-imidazole	Manual	HPLC	180	13% AY	> 99%	n.d	Kamarainen et al. 2004
NITTP	Automated	SPE	50	21% RCY	> 97%	n.d	Kamarainen et al. 2004
NITTP	Automated	SPE	40	51% RCY	> 95%	n.d	Tang et al. 2005
NITTP	Automated	HPLC	60	60% RCY	> 95%	n.d	Tang et al. 2005
NITTP	Manual	HPLC	80	30–40% RCY	n.d	n.d	Adamsen et al. 2005
NITTP	Cassette based	HPLC	70	54% RCY	98,2%	337	Oh et al 2005
NIATP	Automated	SPE	65	30% AY	> 97%	n.d	Chang et al. 2007
NITTP	Automated	SPE	50	55% RCY	> 98%	370	Wang et al. 2009
NITTP	Automated	SPE	40	38% AY	> 95%	n.d	Nandy et al. 2010
NITTP	Microfluidic system	HPLC	60	58% RCY	> 99%	138	Yokell et al. 2012
NITTP	Cassette based	SPE	44	25% AY	> 98%	n.d	Lee et al. 2013
NITTP	Cassette based	SPE	52	41% AY	n.d	n.d	Krcal et al. 2013
NITTP	Automated	SPE	35	50% RCY	> 98%	200	Blom et al. 2014
NITTP	Microfluidic system	HPLC	106	28% AY	> 99%	119	Zheng et al. 2015
(R/S)-1-Epoxy-propyl-2-nitroimidazole	Automated	SPE	120	(R)-: 2% (S)-: 7% AY	n.d	100	Revunov et al. 2015
NITTP	Automated	SPE	21	40% RCY	> 95%	n.d	Fernandez-Maza et al. 2018
Epoxypropyl tosylate, 2-nitroimidazole	Automated	HPLC	75	26% AY	> 99%	110	Ohkubo et al. 2021
NITTP	Automated	SPE	31	56% AY	99,5%	n.d	Cucchi et al. 2022

[#] RCY = radiochemical yield, AY = activity yield (Coenen et al. 2017)

A significant improvement in the radiosynthesis of [^18^F]FMISO was achieved by Lim and Berridge in 1993, who introduced NITTP (1-(2′-nitro-1′-imidazolyl)-2-*O*-tetrahydropyranyl-3-*O*-tosylpropanol), a precursor allowing ^18^F-labeling in one step (Scheme 1) (Lim and Berridge 1993). This radiolabeling strategy includes ^18^F-substitution of a tosyl leaving moiety, rapid removal of the protecting groups and solid phase extraction (SPE) based purification providing [18F]FMISO in 55–80% yield within 50 min radiosynthesis time. Based on this method it was possible to produce [^18^F]FMISO in high activity amounts (> 10 GBq) per single batch. For further optimization, the NITTP precursor was studied under different radiolabeling conditions such as reaction time, temperature and concentration in a manual radiolabeling process by Patt et al. to yield [^18^F]FMISO in up to 86% radiochemical conversion (Patt et al. 1999; Herth et al. 2021).

Another precursor for [^18^F]FMISO radiosynthesis, NIATP (2’-nitro-1’-imidazolyl)-2-*O*-acetyl-3-*O*-tosylpropanol, Scheme 1) was introduced by Cherif et al.; however the radiochemical yield was with 12–18% distinctly lower in comparison with NITTP (Cherif et al. 1994).

A study using two different radiolabeling methods for the radiosynthesis of [18F]FMISO was conducted by the Solin group in 2004, comparing the epoxypropyl tosylate 2-nitroimidazole based approach with the NITTP based method (Kamarainen et al. 2004). By performing a manual radiolabeling procedure, the radiochemical yield was 13% in case of the epoxypropyl tosylate method and 21% in case of NITTP, respectively, with a synthesis time of 180 min for the first and 86 min for the latter. In a similar study, Adamson et al. performed manual radiolabeling of NITTP with 30–40% radiochemical yield (Adamsen et al. 2005).

When an automated radiosynthesizer was used, the NITTP method showed even higher radiochemical yields (34%). The reason for that is supposed in the higher robustness and reproducibility of the automated radiosynthesis and is of importance when large amounts of [^18^F]FMISO are needed. This was a clear demonstration of the advantage of the NITTP precursor and the first published automated radiosynthesis of [18F]FMISO (Kamarainen et al. 2004).

**Fig. 1:**
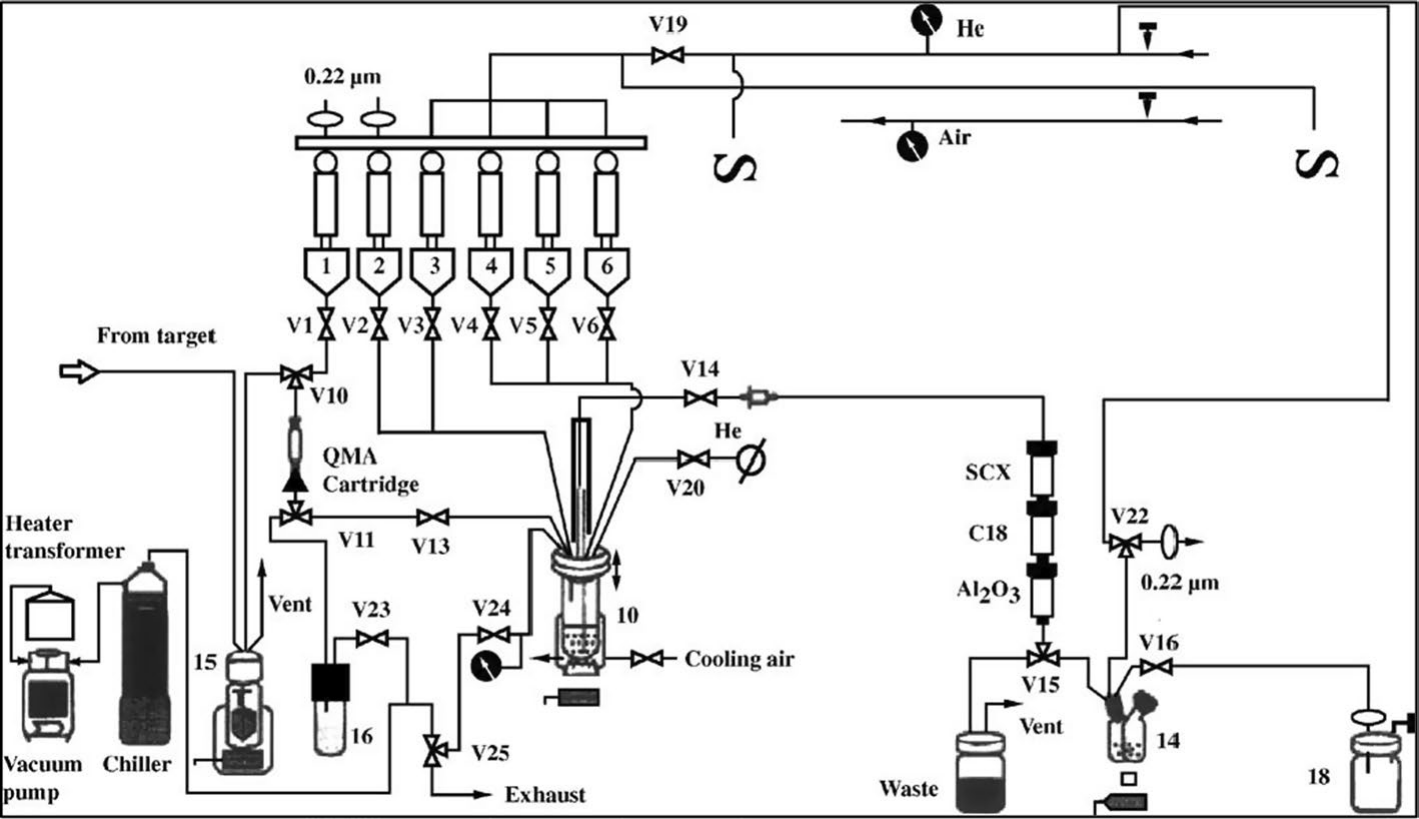
Schematic diagram of automated synthesis of [^18^F]FMISO using Sep-Pak based purification (Tang et al. 2005).

The NITTP based, fully automated one-pot radiosynthesis was further improved by Tang et al., who compared SPE and HPLC purification of [^18^F]FMISO (Tang et al. 2005). In both methods the radiochemical purity of [^18^F]FMISO was greater than 95%, hence the Sep-Pak based approach was realized within shorter radiosynthesis time (40 min vs. 60 min), however, radiochemical yields were slightly lower (51% vs. 60%). (Table 1, Fig. 1).

In the following years the automated radiosynthesis of [^18^F]FMISO was evolved by several groups. All methods are based on SPE separation, which results in a shortened radiosynthesis time and higher radiochemical purity (Chang et al. 2007; Wang et al. 2009; Nandy et al. 2010). Wang et al. presented an automated radiosynthesis procedure yielding [^18^F]FMISO in 50 min with 55% radiochemical yield, > 98% radiochemical purity and high molar activity of 370 GBq/µmol (Wang et al. 2009). Similar results with automated radiosynthesizers and further reduced synthesis time of 35 min, 31 min and 21 min were published recently by several groups (Blom and Koziorowski 2014; Cucchi et al. 2022; Fernandez-Maza et al. 2018).

**Fig. 2:**
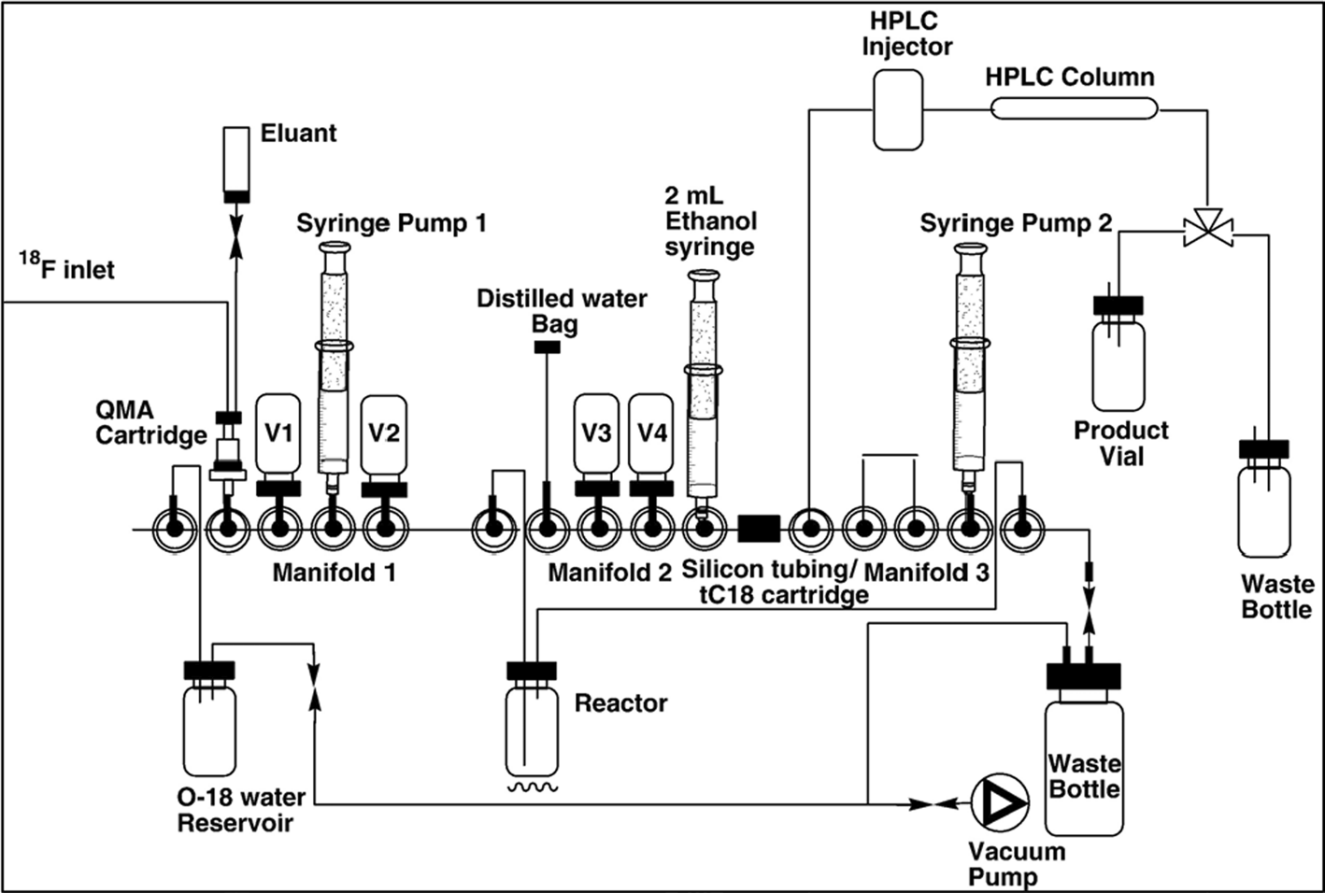
Diagram of the disposable cassette for the radiochemical synthesis of [^18^F]FMISO (Oh et al. 2005).

Cassette-based radiolabeling platforms have gained increasing interest over the last decade due to easy handling and high compliance with GMP regulations (Aerts et al. 2014; Gillings et al. 2021). Consequently, this approach has been also transferred to the radiosynthesis of [^18^F]FMISO. The first radiosynthesis using a disposable cassette was reported in 2005 showing 54% radiochemical yield and high molar activity of 337 GBq/µmol (Oh et al. 2005). The authors used a cassette system designed for [18F]FDG equipped with self-filled glass vials and commercially available cartridges (Fig. 2). However, for final separation a semi-preparative HPLC was still required. In a subsequent publication, the authors applied SPE-based separation which resulted in a reduced synthesis time from previously 70 to 44 min (Lee et al. 2013). Meanwhile a cassette for the [^18^F]FMISO radiosynthesis became commercially available. Krcal et al. reported the results giving a high radiochemical yield of 40% in 52 min synthesis time (Krcal et al. 2013).

**Fig. 3:**
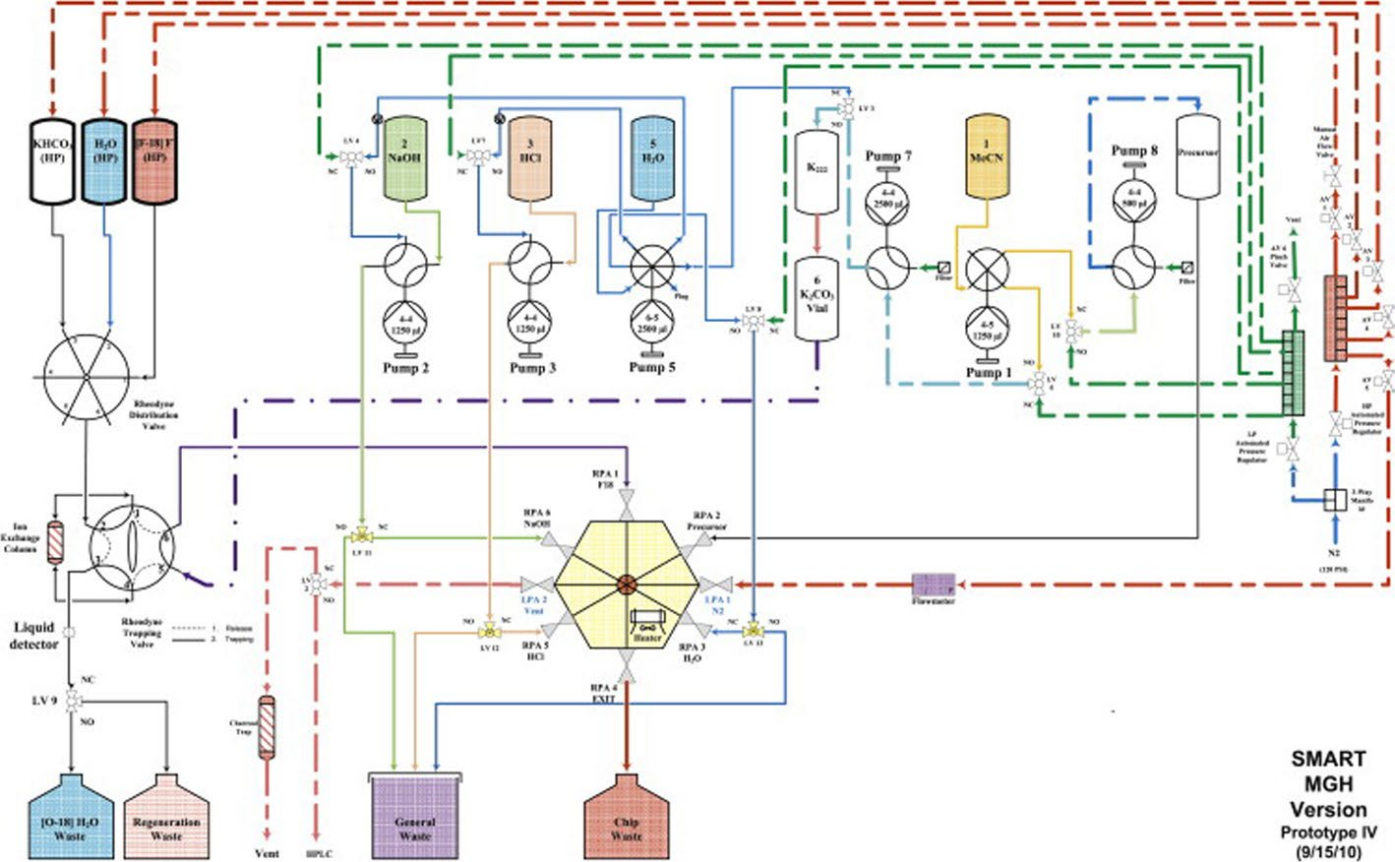
Flow chart of [^18^F]FMISO microfluidic radiochemistry (Yokell et al. 2012).

At the same time syntheses of [^18^F]FMISO on microfluidic systems were reported, however radiochemical yield, purity and radiosynthesis time were not superior to other automated procedures, especially since semi-preparative HPLC for separation and formulation is still necessary (Yokell et al. 2012; Zheng et al. 2015). A flow chart of a microfluidic synthesis unit of [^18^F]FMISO is shown at Fig. 3.

Since [^18^F]FMISO is a chiral compound and clinically used as a racemic mixture, it was of specific interest to radiosynthesize the individual (*R*)- and (*S*)-enantiomers and compare their PET imaging characteristics. The radiosynthesis for that challenging task started from the individual (*R*)- and (*S*)-isomers of the 1-epoxy-propyl-2-nitroimidazoles as precursors. In an automated procedure via a cobalt-complex mediated ring opening with [^18^F]HF the individual [^18^F]FMISO isomers were synthesized with high enantiomeric excess (> 99%) in 2% and 7% radiochemical yield, respectively (Revunov et al. 2015). The (*R*)- and (*S*)-[^18^F]FMISO enantiomers were studied by micro-PET/CT in tumor bearing mice, however the tumor-to-muscle ratios were similar, although the (*R*)-enantiomer showed a somewhat faster initial tumor uptake.

In a very recent work the epoxypropyltosylate based ^18^F-radiofluorination to radiosynthesize [^18^F]FMISO was reinvestigated. By using an automated system and SPE-based separation, a radiochemical yield of 26% was achieved within 75 min radiosynthesis time (Ohkubo et al. 2021).

At Helmholtz-Zentrum Dresden-Rossendorf (HZDR) we have set up a feasible and GMP compliant radiosynthesis based on commercially available cassettes for the FASTlab system for use of [^18^F]FMISO in clinical applications. Using this method, [^18^F]FMISO can be produced in high radiochemical yield and pharmaceutical quality according to the requirements of the European Pharmacopoeia.

However, as [^18^F]FMISO apart from patient supply was also required for preclinical studies, we investigated a method combining the reliable cassette based automated procedure with a cost-saving modification. As result, we used the FASTlab radiosynthesizer, but performed the radiosynthesis with in-house prepared cassettes, in an approach similar to that of Oh et al. ^19^. In the experimental part of this study we compared our results obtained with in-house prepared FASTlab cassettes for the [18F]FMISO radiosynthesis with the commercially available cassettes in terms of radiochemical yield, reliability and results of quality control.

## Methods

### General

All radiochemical yields (RCY) presented are decay corrected. Commercial cassettes for [^18^F]FMISO FASTlab syntheses were purchased from GE HealthCare (Braunschweig, Germany). For automated radiosynthesis, a FASTlab1 radiosynthesizer (GE HealthCare) was used. Fluoromisonidazole as reference standard and the NITTP precursor was purchased from ABX GmbH (Radeberg, Germany). Electrolyte solution E153, pH 5.0–7.0, containing sodium chloride (5.26 g/L), potassium chloride (0.37 g/L), calcium chloride dihydrate (0.37 g/L), magnesium chloride hexahydrate (0,3 g/L) and sodium acetate trihydrate (6.8 g/L) was purchased from Serumwerk Bernburg AG (Bernburg (Saale), Germany). Oasis QMA light cartridges (carbonate form), Oasis HLB cartridges and alumina N cartridges were supplied from Waters (Eschborn, Germany) Tetrabutylammonium hydrogencarbonate was purchased from Enamine (Kyiv, Ukraine). Acetonitrile, ethanol, and phosphoric acid were supplied by Sigma-Aldrich/Merck (Darmstadt, Germany). Water for injection (100 ml plastic bags) was purchased from B. Braun (Melsungen, Germany) Rubber stoppers and crimp caps for vials were purchased from Isera (Düren, Germany). [^18^F]Fluoride was produced by bombardment of H_2_[^18^O]O (Rotem, Israel) in an niobium target using the in-house installed TR-Flex cyclotron (ACSI, Vancouver, Canada). For measurement of radioactivity, an ISOMED 2010 activimeter (Nuklearmedizintechnik Dresden, Germany) was used. Analytical HPLC was performed by an AGILENT 1200 system (Waldbronn, Germany) equipped with an UV- and radioactivity detector (GABI. Raytest, Germany). Gas chromatography analyses were performed with a GC/MS 7890 system (AGILENT, Waldbronn, Germany). Radio-thin layer chromatography (radio-TLC) was performed with a CR35Bio TLC scanner (Elysia-Raytest, Belgium).

### Automated radiosynthesis of [^18^F]FMISO using original FASTlab cassettes

**Fig. 4:**
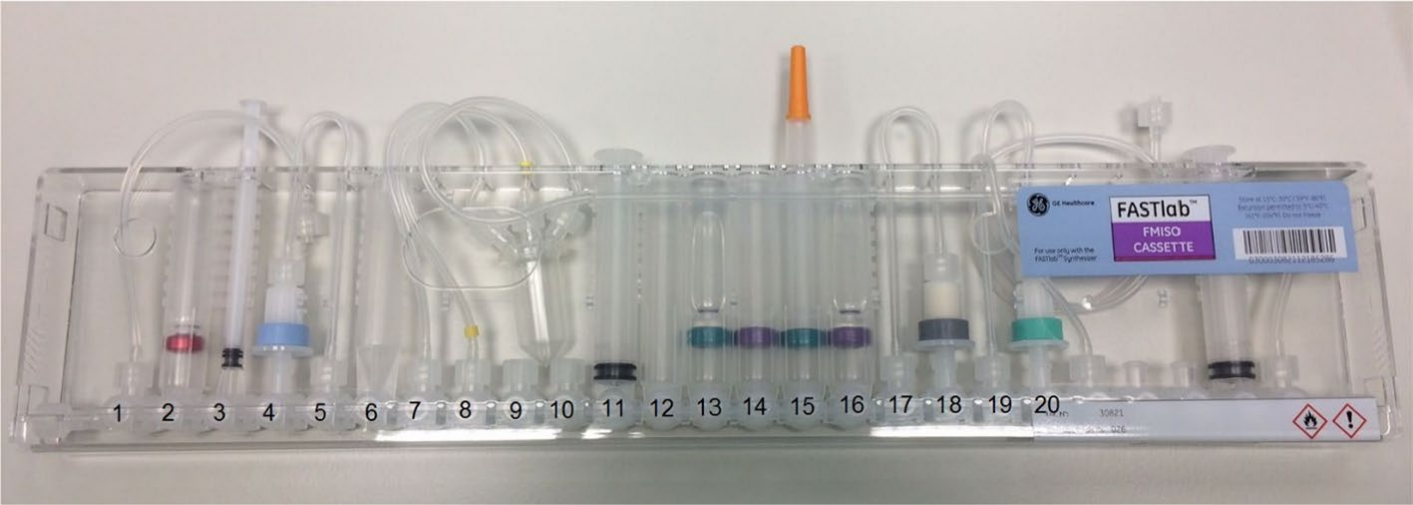
FASTlab cassette for [^18^F]FMISO, for reagents see Table 2

Radiosynthesis of [^18^F]FMISO with the FASTlab system was performed according to the manufacturers advice (FASTlab FMISO Application Manual, GE Healthcare DOC1705995). The commercially available [^18^F]FMISO cassette contains all required reagents, solvents and cartridges, with exception of the NITTP precursor. This has to be stored separately in a refrigerator at − 18 °C and has to be inserted into the cassette shortly before the start of radiosynthesis. A FASTlab cassette for [^18^F]FMISO is displayed in Fig. 4.

The lab unit test checks the correct function of pressure and vacuum regulators, the presence of adequate nitrogen gas and compressed air pressure and the mechanical function of the radiosynthesizer. After successful lab unit test the cassette for [18F]FMISO is mounted into the radiosynthesizer. The vial with the NITTP precursor (7 mg, 16.4 µmol) that is stored in powder form in the refrigerator has to be inserted in the cassette prior installation on the radiosynthesizer. After adding a water bag, placing the reaction vial in the heating element and connecting the tube lines for product collection and target water recovery the cassette test is performed. During the cassette test the gas and vacuum tightness of the cassette is checked, the vials are punched and the cartridges are conditioned by elution with water and solvents. After the cassette test was successful, the system is ready for receiving the [^18^F]fluoride solution. The setup time for system and cassette test is approximately 20 min.

**Scheme 2.**
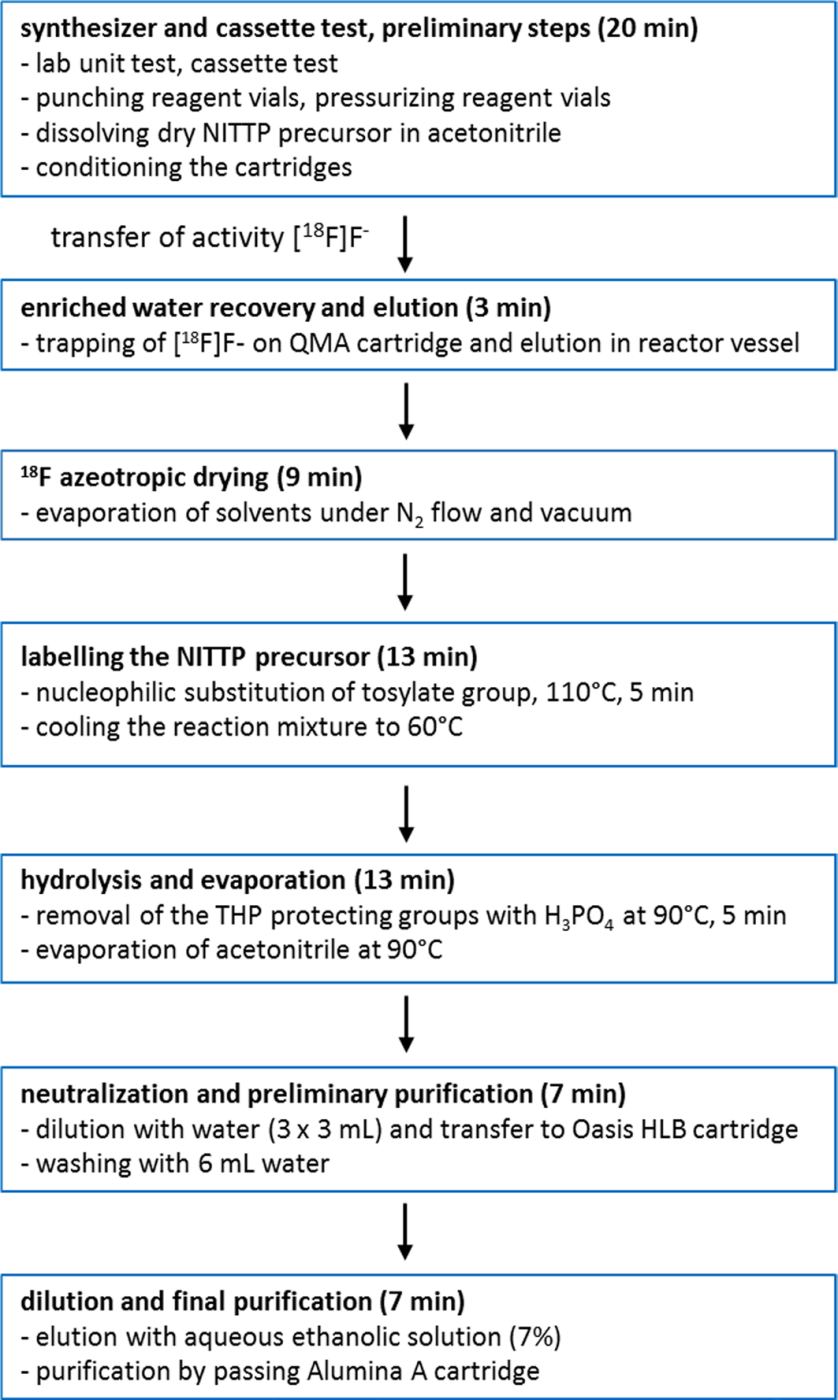
Flow chart of all steps of [^18^F]FMISO synthesis with FASTlab

After receiving the activity, the radiosynthesis runs fully automated. The radiosynthesis time is about 48 min. The flow chart of the following [^18^F]FMISO synthesis steps with FASTlab is displayed in Scheme 2.

After finishing the radiosynthesis, [^18^F]FMISO is eluted in a separate product collection vessel, the product volume delivered by the radiosynthesizer is 20 mL. For dispensing and sterile filtration [^18^F]FMISO was transferred to a dispensing unit in a separate hot cell.

After discharging the radiotracer, the FASTlab synthesizer performs a rinsing procedure to send remaining activity to the waste bottle, which takes about 10 min time.

### Automated radiosynthesis of [^18^F]FMISO using in-house prepared FASTlab cassettes

Before a used cassette can be prepared, it has to be emptied from all vials and cartridges, after decay of radioactivity. The reaction vessel is removed, the manifold and all tube lines are flushed to dryness in a nitrogen stream whereas all 3-way valves of the manifold must be turned. The plunger of the 1.0 mL syringe (position 3) should be replaced. The reaction vessel is washed separately with acetone, dried under a vacuum line and connected to the cassette on its original position. The glass vials for solvents and reagents are cleaned and dried after removal of the crimp caps. After filling with new reagent or solvent, the vials must be sealed with new rubber stoppers and crimp caps.

The dried cassette is equipped with the starting materials as displayed in Table 2 at the indicated positions (see Fig. 4). In addition, the starting material used in the original cassette is shown.

**Table 2:** Starting materials used in in-house prepared and original cassettes for [^18^F]FMISO FASTlab

Position	In-house prepared cassette	Original cassette
1	*Unaltered*	
2	Eluent, (acetonitrile/150 mM N(Bu)_4_HCO_3_ 75:25), (1.0 mL)	Mixture (0.8 mL)
3	*Unaltered*	
4	SepPak, QMA light cartridge, carbonate form, 130 mg	SepPak, QMA light cartridge, carbonate form
5	*Unaltered*	
7–8	*Unaltered*	
11	*Unaltered*	
12	Precursor (NITTP, 10 mg), dissolved in acetonitrile (0.2 mL)	Precursor (NITTP, 7 mg), powder
13	Acetonitrile (4.0 mL)	Acetonitrile (4.2 mL)
14	Phosphoric acid 2.3 M (4.0 mL)	Phosphoric acid (4.2 mL)
15	*Unaltered*	
16	Ethanol 95% (4,0 mL)	Ethanol (4,0 mL)
17	*Unaltered*	
18	Oasis HLB cartridge (225 mg)	Oasis HLB cartridge
19	*Unaltered*	
20	SepPak Alumina B cartridge (280 mg)	SepPak Alumina A cartridge

The sealed vials were inserted into the cassette slots, but not punched yet. The SepPak QMA light, OASIS HLB and SepPak Alumina B cartridges were tightly connected with the manifold and tubing without previous activation. In comparison to the original FASTlab cassette, an Alumina B cartridge was used leading to same results.

Since NITTP precursor is commercially available in 10 mg units we decided to avoid a portioning process into 7 mg (16.4 µmol), and dissolved the whole 10 mg in 200 µL acetonitrile before transferring the solution to the vial, suitable for the cassette. In addition, we performed also three experiments with 5 mg (11.7 µmol) NITTP, however the radiochemical yield of [^18^F]FMISO turned out to be significantly lower in this case.

After inserting the in-house prepared cassette in the FASTlab synthesizer and plugging in the water bag and all other connections, the radiosynthesis of [^18^F]FMISO was performed by the identical procedure as described for the commercial cassettes.

## Results

The radiochemical yield of [^18^F]FMISO using original cassettes for FASTlab was 49 ± 7% (n = 30) within 48 min synthesis time. A radiochemical purity of 99.9% and a molar activity of 565 GBq/µmol based on [^18^F]Fluoride starting activity of 56 GBq was reached. All specifications according to the Ph. Eur. monograph for [^18^F]FMISO (2459) were met, including radionuclidic purity, sterility and content of endotoxins (see Table 3), with exception of the pH value. This was found to be 4.5 for the [^18^F]FMISO solution delivered from the FASTlab synthesizer. Adjustment to pH = 6.2 was achieved by addition of 2 mL of electrolyte solution for injection (E153, pH = 5–7) to the final batch, resulting in a final product volume of 22 mL. The formulated [^18^F]FMISO solution was sterile filtered. The quality parameter are displayed in Table 3.

**Table 3:** Results and typical values of [^18^F]FMISO injection solution obtained with FASTlab cassettes

	Specification according to Ph. Eur. monograph 2459	[^18^F]FMISO specification (GE Healthcare DOC1705995)	Found with original FASTlab cassette	Found with in-house prepared FASTlab cassette
Radiochemical yield d.c	Not specified	≥ 26%	49% (n = 27)	39% (n = 19)
Radiochemical purity [^18^F]FMISO (HPLC)	≥ 95%	> 95%	99.9%	99.8%
Radiochemical purity [^18^F]F^−^ (TLC)	≤ 5%	not specified	0.3%	0.1%
Content of FMISO (HPLC)	≤ 10 µg/mL**	< 5 µg/mL	0.3 µg/mL	0.2 µg/mL
Content of DH-MISO (HPLC)	≤ 10 µg/mL**	< 5 µg/mL	1.0 µg/mL	0.8 µg/mL
Highest content of single other impurity (HPLC)	≤ 10 µg/mL**	< 5 µg/mL	0.8 µg/mL	2.7 µg/mL
Total content of chemical impurities (HPLC)	≤ 50 µg/mL**	< 10 µg/mL	3.6 µg/mL	6.8 µg/mL
TBA	≤ 260 µg/mL**	< 170 µg/mL	< 260 µg/mL	< 260 µg/mL
Radionuclidic purity (half-life)	1.75–1.92 h	1.75–1.92 h	1.84 h	1.84 h
Ethanol (GC–MS)	< 10% v/v	< 7.9%	6.0%	6.0%
Residual solvents acetonitrile (GC–MS)	< 410 µg/mL**	< 273 µg/mL	27 µg/mL	26 µg/mL
pH	4.5–8.5	4.5–8.0	6.2*	6.8*
Bacterial endotoxins (LAL)	< 175 IU/mL	< 175 IU/mL	< 0.8 IU/mL	< 0.8 IU/mL
Molar activity (HPLC)	Not specified	Not specified	565 GBq/µmol	588 GBq/µmol

*After addition of 2.0 mL E153 solution for injection

**Based on a maximum dose volume of 10 mL

The radiochemical yield of [^18^F]FMISO obtained with in-house prepared cassettes and 10 mg (23.5 µmol) NITTP precursor was 39 ± 11% (n = 19), which is somewhat lower in comparison with the original cassettes. The radiochemical and chemical purity and all other parameters of quality control are identical with [^18^F]FMISO obtained from original cassettes (Table 3). As already mentioned, when using 5 mg (11.7 µmol) NITTP precursor the radiochemical yield of [^18^F]FMISO was 26% only (n = 3).

## Discussion

With view on the results in Table 3 it can be summarized, that the radiosynthesis with original FASTlab cassettes provides [^18^F]FMISO in high radiochemical yield and excellent pharmaceutical quality and all specifications of the Ph. Eur. monograph 2459 are met. The activity amount of [^18^F]FMISO obtained by this method with original FASTlab cassettes is in the range of 5–35 GBq, starting from 20 to 105 GBq of [^18^F]fluoride respectively, resulting in an activity concentration of 220–1590 MBq/mL at end of synthesis.

The radiosynthesis of [^18^F]FMISO with in-house prepared FASTlab cassettes is successful when the following requirements are fulfilled. First, the careful and rigorous drying of the manifold, the tube lines and the reaction vial of the cassette is mandatory. This is done by flushing with a nitrogen stream for 2 min. The reaction vessel was additionally dried under vacuum. The second important point is the sealing of the glass vials containing reactants and solvents with stoppers and crimp caps. The rubber stopper should have adequate thickness to guarantee tight closure, but must show additionally appropriate quality for the puncher of the radiosynthesizer. Thin rubber stoppers causing leakage of the vial after punching, thick rubber material may resist the punching.

[^18^F]FMISO produced with in-house prepared FASTlab cassettes shows, beside of total impurities, similar values for quality control specifications as compared with the original cassettes. Hence, the radiochemical yields were on average 10% lower, but still high enough to be within the specification of the cassette supplier (GEHC). The content of highest single other impurity and the content of total chemical impurities were in the in-house prepared cassette increased by factor 2 and 3, respectively (Table 3). This may be explained by the use of research grade chemicals instead of GMP grade substances apart from the NITTP precursor. The use of 10 mg NITTP precursor instead of 7 mg in the original cassettes did not have significant impact on radiochemical yield and quality of the produced product. The pH was adjusted in both types by addition of 2.0 mL buffer solution (E153) to the final [^18^F]FMISO solution. The molar activity achieved was > 500 GBq/µmol for both type of cassettes what surpasses all data from the literature (see Table 1).

**Fig. 5:**
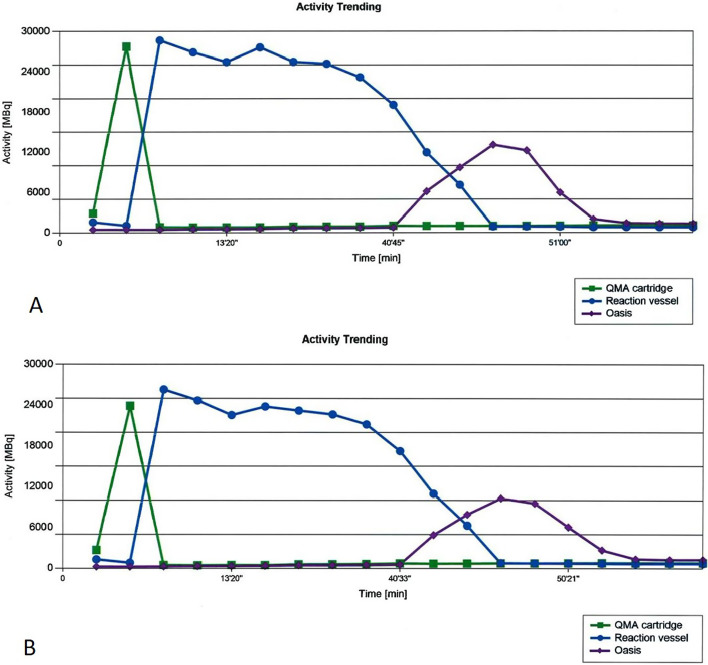
**A** Activity trending of [^18^F]FMISO FASTlab radiosynthesis with original cassette, **B** Activity trending of [^18^F]FMISO FASTlab radiosynthesis with in-house prepared cassette

The course of radioactivity during the radiosynthesis of [^18^F]FMISO is recorded by radioactivity detectors installed within the radiosynthesizer and is displayed as “activity trending” at the end of synthesis. A graphic overview of this activity trending for the original cassette and for the in-house prepared cassette is displayed in Fig. 5. An almost identical track in both figures proves the conformity of both techniques.

## Conclusion

[^18^F]FMISO is a highly clinical relevant radiopharmaceutical for imaging of hypoxia with PET. Since its introduction more than 30 years ago, radiopharmaceutical chemists’ efforts have increased to make the [^18^F]FMISO radiosynthesis more simple and convenient. Today, a number of automated, cassette-based and GMP compliant methods are available, assuring the broad availability of the tracer as radiopharmaceutical so that the clinical demands can be covered. In this study we could demonstrate that [^18^F]FMISO can be produced with the FASTlab system in high radiochemical yield (49%) in excellent radiochemical purity (99.9%) and high molar activity (> 500 GBq/µmol). Furthermore we investigated the [^18^F]FMISO radiosynthesis with in-house prepared FASTlab cassettes, to have a resource for pre-clinical investigation, such as small animal PET/CT imaging. As we have found, the use of in-house prepared FASTlab cassettes is a favorable option, yielding [^18^F]FMISO in a slightly diminished radiochemical yield (39%), comparable pharmaceutical quality, and a cost reduction of 70% in comparison to the original cassette. Commercially available cassettes are the preferred choice for clinical application, while in-house prepared cassettes present an interesting and well-priced option to get [^18^F]FMISO easily available for research purposes.

## Data Availability

All data generated or analysed during this study are included in this published article.

## Abbreviations

AY: Activity yield
CT: Computed tomography
DH-MISO: 3-(2-Nitro-1*H*-imidazol-1-yl)propane-1,2-diol (Desmethylmisonidazole)
GC: Gas chromatography
GMP: Good manufacturing practice
HPLC: High-performance liquid chromatography
MRI: Magnetic resonance imaging
MS: Mass spectrometry
NIATP: (2′-Nitro-1′-imidazolyl)-2-*O*-acetyl-3-*O*-tosylpropanol
NITTP: 1-(2′-Nitro-1′-imidazolyl)-2-*O*-tetrahydropyranyl-3-*O*-tosylpropanol
PET: Positron emission tomography
Ph. Eur.: European pharmacopoeia
RCY: Radiochemical yield
SPE: Solid-phase extraction
SPECT: Single photon emission computed tomography
TBA: Tetrabutylammonium
TLC: Thin layer chromatography
[^18^F]FMISO: 1*H*-1-(3-[^18^F]fluoro-2-hydroxypropyl)-2-nitroimidazole
